# The Effect of Steel and Polypropylene Fibers on the Properties of Horizontally Formed Concrete

**DOI:** 10.3390/ma13245827

**Published:** 2020-12-21

**Authors:** Adrian Chajec, Łukasz Sadowski

**Affiliations:** Department of Building Engineering, Wroclaw University of Science and Technology, Wybrzeże Wyspiańskiego 27, 50-370 Wroclaw, Poland; lukasz.sadowski@pwr.edu.pl

**Keywords:** polypropylene fibers, steel fibers, concrete floors

## Abstract

The article presents a comparative analysis of the impact of the addition of steel and polypropylene fibers on the properties of the concrete mixes and hardened concrete used in the concrete floor industry. The behavior of concrete intended for floors is different from conventional structural concrete because it is formed horizontally; until now, the effect of steel and polypropylene fibers on the properties of concrete formed horizontally has not yet been fully understood. Therefore, the aim of this article is to examine this issue and compare the behavior of concrete modified with steel and polypropylene fibers in concrete that is formed horizontally. The following properties of fresh concrete mixes were analyzed: consistency, the content of air-voids, and bulk density. Consequently, the following properties of hardened concrete were analyzed: compressive strength, bending tensile strength, and brittleness. It was confirmed that steel and polypropylene fibers have a different type of effect on the properties of fresh concrete mixes and hardened concrete. Finally, a combined economic and mechanical analysis was performed.

## 1. Introduction

Horizontally formed concrete is commonly used in civil engineering. It is especially used in floors, which are one of the most important construction elements in buildings. An important problem regarding horizontally formed concrete is the heterogeneity of its mechanical properties. Previously conducted research [1] shows that the compressive strength of concrete changes across the cross-section of a concrete element. This results in the low compressive strength of the concrete in the upper part of its cross-section (about 30–40% of the designed strength of concrete). According to research [2], the heterogeneity of concrete also causes the delamination and loosening of its top layer, which contributes to its damage. Fibers can improve the homogeneity of the concrete.

Floors are very often very expensive and difficult to make [1,2]. In order to make a durable floor, many factors should be investigated. Concrete floors often consist of several layers [3]. In industrial concrete formed horizontally, the surface of the floor is the most important [4]. The durability of the overlay is strongly related to the properties of the concrete that was used to execute the floor [3,5]. To prevent any defects and damage, the concrete mix should be specially designed and investigated.

The properties of concrete mainly depend on the ingredients of the concrete mix and the used additives [6,7,8]. To prevent early age cracking, the concrete that is used to make floors is frequently modified with the addition of fibers [9,10], which are usually made of steel and polypropylene [11,12].

Concrete formed horizontally is usually reinforced with reinforcement bars or meshes; however, the method of reinforcing these elements differs significantly from other structural elements (such as columns, beams) [13]. Nevertheless, frequent damage to floors due to concrete shrinkage is observed [14,15]. It is believed that the use of only reinforcing bars will not protect the floor from cracking caused by concrete shrinkage. In order to reduce the occurrence of cracks, the addition of fibers is used [16,17]. However, it should be emphasized that using fibers instead of bar reinforcements is not effective and can be seen to be a mistake [18,19]. It is best to use bars for mechanical reasons and the addition of fibers for protecting against early age cracking. The main function of the fibers in concrete is to reduce the initiation and propagation of cracks [20]. Fiber reinforcement can increase the energy absorption and impact strength of concrete [21,22]. The main task of fibers is to improve the properties of a concrete floor and not to improve the construction conditions of the element [23]. Fibers have a significant impact on the properties of concrete mixes and hardened concrete. However, designing concrete mixes with the addition of fibers is a difficult task. Different types, shapes and dimensions of fibers have a significant impact on concrete. It was presented (e.g., in [24]) that the application of fibers with hooked ends is crucial when improving the energy absorption capacity of a sustainable ultra-high-performance fiber-reinforced concrete in quasi-static mode. In order to design durable and resistant concrete floors with the addition of fibers, research must be carried out. In many cases (e.g., in [24]), this research can be analyzed with regards to just one type of fiber, but more frequently, the comparison of two types of fibers should be performed (e.g., like in [25] in order to analyze the flexural performance of the obtained material).

The behavior of concrete designed for floors is different from that of conventional structural concrete [26,27]. To date, the effect of steel and polypropylene fibers on the properties of concrete formed horizontally has not been fully understood. Therefore, the aim of this article is to examine this issue and compare the behavior of concrete modified with steel and polypropylene fibers in floors. To achieve this goal, the fundamental properties of fresh concrete mixes and hardened concrete modified with steel and polypropylene fibers were analyzed. To test the effect of fibers on floor concrete, it was decided to investigate the addition of steel fibers in the amount of 20–32.5 kg/m^3^. In order to estimate the influence of these fibers on the concrete, it was decided to add the fibers while repeatedly increasing their dosage by 2.5 kg/m^3^. It should be emphasized that a lower addition of steel fibers (20–30 kg/m^3^) is used in floor concretes than in construction concretes (40–80 kg/m^3^) [28,29]. Nowadays, there is a trend related to the search for the possibility of recycling plastic waste materials [30,31]. One such example is the use of plastic as an additive to concrete in the form of polypropylene fibers. The producers of these fibers propose an additive in the amount of 2.5–3.0 kg/m^3^ (product dedicated to floor concretes). To investigate the effect of the addition of these fibers on the concrete mix and hardened concrete, test series were carried out with the addition of 1.5–4.0 kg/m^3^ fibers (repeatedly increasing their dosage by 0.5 kg/m^3^). It should be emphasized that the polypropylene fibers used in these tests are not commonly used in construction concretes. Out of the properties of a fresh concrete mix, special focus was placed on the consistency, air-void content, and bulk density. In order to analyze the mechanical properties of the concrete, compressive strength and bending tensile strength tests were carried out. On the basis of the above research, an analysis of the mechanical and economic properties of the concrete with the addition of fibers was performed.

## 2. Materials and Methods

### 2.1. Materials Used in the Research

**Figure 1:**
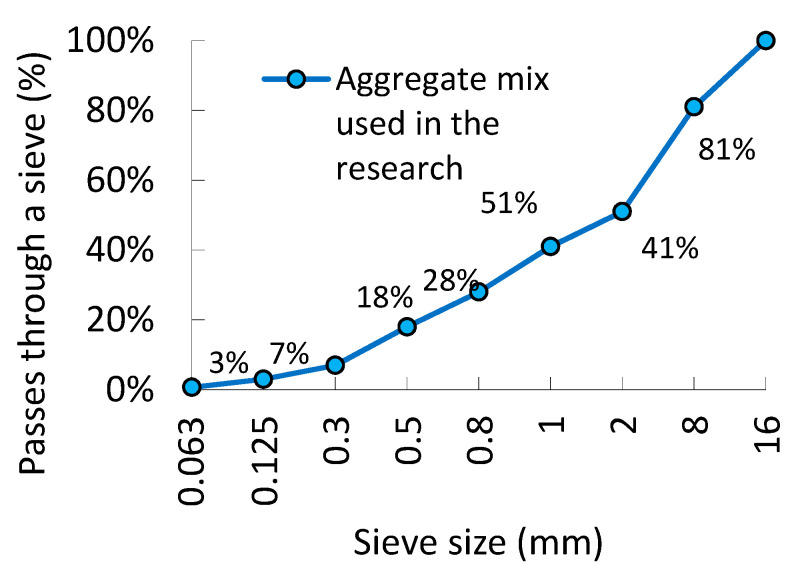
The screening curve of the aggregates used in the tests.

**Figure 2:**
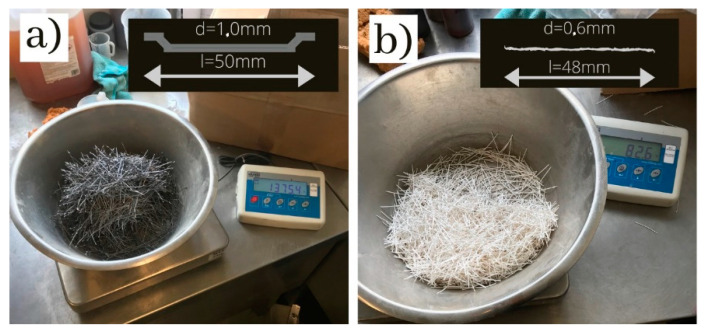
Fibers used in the research (**a**) steel fibers, (**b**) polypropylene fibers.

Concrete of class C25/30 with a fixed composition was subjected to research. The tested series differed only in the content and type of fibers. In the research, CEM III 42.5 N cement (320 kg/m^3^, Buzzi Unicem, Hranice, Czech Republic) and potable water (w/c = 0.5 (-), which was added to the concrete mixes, were used. Figure 1 shows the screening curve of the aggregate that was used in the tests (sand 0–2 mm 700 kg/m^3^, gravel 2–8 mm 443 kg/m^3^, gravel 8–16 mm 700 kg/m^3^—all the aggregates came from Kopalnia Byczeń, Byczeń, Poland). Two different types of fibers were added to the concrete mix: type 1—steel fibers with both ends hooked (Bautech, Piaseczno, Poland, length: 50 mm, diameter: 1.0 mm, tensile strength: 1100 MPa, Young’s modulus: 180 GPa, density: 7650 kg/m^3^); type 2—continuous polypropylene fibers (Ha-Be, Łozina, Poland, length: 48 mm, diameter: 0.6 mm, tensile strength: 600 MPa, Young’s modulus: 5 GPa, density: 910 kg/m^3^). The fibers used in the research are shown in Figure 2. Superplasticizer Pantarhit FM (Ha-Be, Łozina, Poland, 3.84 kg/m^3^) was added to the mix.

### 2.2. Preparation of Samples

The prepared dry components of the concrete mix (cement, fine and coarse aggregate, fibers) were measured (weighed) and then placed together in a mixer (TECHKAZ, Brodnica, Poland). Pre-mixing was then performed for 90 s. After this, about 2/3 of the volume of water was added to the mix. After mixing for about 60 s, the mechanical mixing was stopped, and the ingredients were mixed manually to avoid lumps. Stirring was then continued, and the superplasticizer was added with the remaining 1/3 volume of water. After the addition of all the ingredients, the mixture was mixed for an additional 60 s. After the mixing, tests of the fresh concrete mix were performed. After testing the fresh mix, the samples were formed. The mixture (in two layers) was placed into the previously prepared molds (covered with an anti-adhesive substance) and then compacted until any visible air bubbles were removed (about 60 s). After molding, the samples were left for 24 h. After this time, the samples were removed from the molds and then stored in water curing conditions. After the specified time (28 days), nondestructive and destructive tests were performed. Table 1 shows the concrete mix series that was prepared to conduct the research.

### 2.3. Determination of the Properties of the Fresh and Hardened Concrete Mixes

In order to determine the properties of the fresh mixes, the tests were performed immediately after the mixing procedure. In turn, to investigate the properties of the hardened concrete mixes, the tests were conducted after 28 days of molding the specimens.

#### 2.3.1. Consistency of the Fresh Concrete Mixes

In order to determine the consistency of the concrete mixes with the addition of fibers, a “standard bucket” method was used. In this method, a bucket was placed on a prepared steel plate (GEOLAB, Warszawa, Poland) (covered with a release agent) and filled to 1/3 of its height. Afterward, the concrete mix was compacted with a rod (12 strokes), and the bucket was filled with the concrete mix to 2/3 of its height and compacted again. Finally, the bucket was completely filled and compacted. After this, the surface of the concrete mix was leveled, and the bucket was slowly but firmly lifted. The distance between the fallen cone of the concrete mix and the top of the empty bucket, which was placed on a steel plate, was measured. The test was carried out in accordance with the procedure described in the standard [32].

#### 2.3.2. Air-Void Content in the Fresh Concrete Mixes

The investigation of the air content was carried out according to the water column method. It involved preparing an air-test container, placing the concrete mix inside it (up to 1/2 of its height), and then vibrating it. After this, the next part of the concrete mix was added (in order to fill the entire container) and vibrated. The surface of the mix was then be leveled, the lid was being put on, and the locks were closed. Afterward, the device was filled with water through one of the valves until the water overflowed through the other valve, and then both valves were closed. The next step involved an air pump, with the use of which the air was pumped in order to increase the pressure inside the device. After reaching the maximum pressure on the manometer, a button on the lid was pressed to equalize the pressure in the device. The reading on the manometer after this operation showed the amount of air in the concrete mix. The test was carried out in accordance with the standard [33].

#### 2.3.3. Bulk Density of the Fresh and Hardened Concrete Mixes

The determination of the bulk density of the fresh concrete mixes consists of placing the mixture in a vessel with a known volume and weight, which is then weighed. The bulk density of the fresh concrete mix is then calculated. The determination of the bulk density of the hardened concrete consists of drying the sample to a constant weight and then weighing the sample. The sample should also be carefully measured to determine its volume. Based on the measurements, the bulk density of the hardened concrete is calculated. In order to determine the bulk density of the concrete with the addition of fibers, the tests were conducted according to the standard [34].

### 2.4. Determination of the Mechanical Properties of the Hardened Concrete

In order to determine the mechanical properties of the concrete with the addition of fibers, destructive tests (concrete compressive strength test and concrete bending tensile strength) were conducted exactly after 28 days after molding the specimens. Tests of the compressive strength of the concrete were performed on cubic samples (15 cm × 15 cm), whereas tests of the bending tensile strength of the concrete were performed on beam samples (15 cm × 15 cm × 60 cm). The compressive strength was investigated using an endurance machine (HGM Maszyny, Chorzów, Poland). After the storage time (28 days), the samples were placed in the jaws of the machine, and then the load was applied at normal speed until the sample was destroyed. The destructive force was recorded. The test was performed on at least 6 samples of the same test series. The bending tensile strength was also investigated using an endurance machine. Samples were placed in special jaws (two points of support, two points of application of load), and then the load was applied at the standard speed until the sample was destroyed. The destructive force was again recorded. The test was performed on at least 4 samples of the same test series in order to ensure the appropriate number of results for statistical purposes. All the tests were performed in a laboratory at Wroclaw University of Science and Technology. Based on the conducted destructive tests, the value of brittleness (B) was determined according to Equation (1):

(1) $$B=\frac{f_{\mathrm{ctm}}}{f_{\mathrm{cm}}}\;\left(-\right).\;$$

where: *f*_ctm_—the bending tensile strength [MPa], *f_cm_*—the compressive strength of the concrete [MPa].

Brittleness is an indicator that describes how the destruction of material looks under the influence of an acting load. The higher the brittleness, the more the concrete is damaged in an unsigned and catastrophic manner (for example, a single crack or a small number of large cracks appear). In turn, the lower the brittleness, the more visible the damage in the concrete is (a large number of cracks occur with a smaller width). Generally, brittleness B is a parameter that describes the relationship between the basic mechanical properties of concrete. If the brittleness ratio is greater than 0.125 (–), then the concrete is very brittle.

## 3. Results and Analysis

### 3.1. Determination of the Properties of the Fresh Concrete Mixes and Hardened Concrete

#### 3.1.1. Consistency of the Concrete Mixes

**Figure 3:**
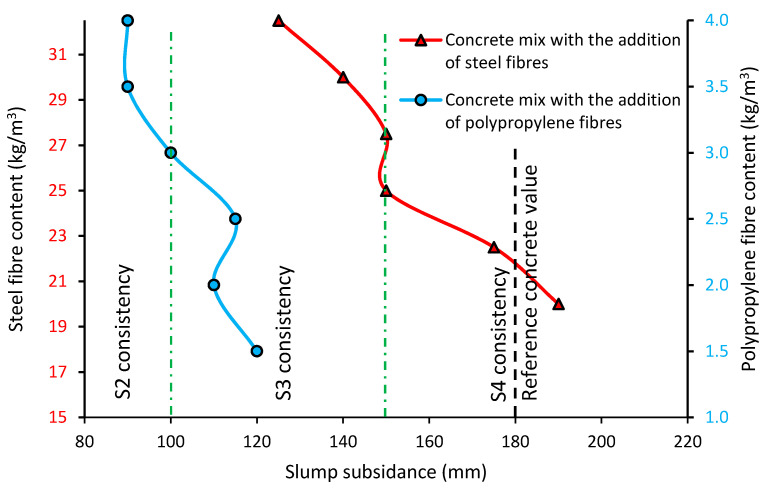
Slump subsidence of the concrete mixes modified with the addition of fibers.

Figure 3 presents the slump subsidence value for the concrete mixes with the addition of steel (red line) and polypropylene (blue line) fibers. The results were compared with the slump subsidence value of the reference concrete mix. The green vertical lines indicate the different consistency classes of the concrete.

Figure 3 shows the slump subsidence value of the concrete mixes with different types and contents of fibers. Generally, the consistency of the concrete mixes modified with the addition of fibers is denser when compared to the reference concrete (even 2 class differences S4–S2). Additionally, the consistency of the mixes modified with fibers changes when the content of fibers increases. The consistency of the concrete mixes with polypropylene fibers is denser when compared to the concrete mixes with steel fibers (on average, there is a 1 consistency class difference). The adding of even 1.5 kg/m^3^ of polypropylene fibers changes the consistency of the concrete mixes from the S4 consistency class to S3. Similar results were achieved in research [35]. The addition of polypropylene fibers also made the consistency denser, whereas the addition of steel fibers caused a slight change in the consistency of the slump (20 mm less subsidence).

Differences in the consistency of the concrete mix modified with fibers may be related to the absorption of water by the fibers. The surface of fiber during mixing requires being surrounded by the cement paste, which reduces the amount of free water in the mix. It can also be caused by the accumulation of fibers—the shape of the fibers causes them to be stuck, and therefore there is an irregular distribution of the number of fibers in the concrete mix. The addition of fibers to the concrete mix also causes them to wedge against the aggregate grains, which prevents their free arrangement in the concrete mix. Some researchers investigated the impact of the length or shape of fibers on the consistency of concrete mixes [36]. Longer fibers have a bigger effect on the consistency of the concrete mix (the consistency is denser). If the shape of the fibers is more irregular, the consistency of concrete is denser.

#### 3.1.2. Air-Void Content in the Concrete Mixes

**Figure 4:**
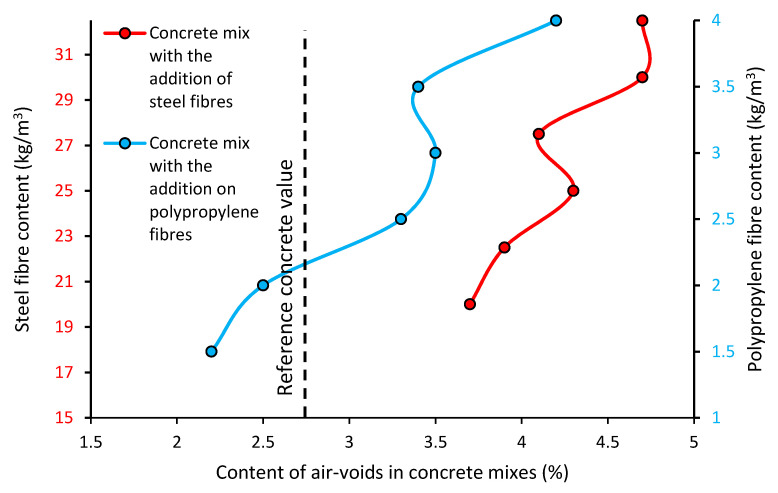
The content of air-voids in the concrete mixes modified with the addition of fibers.

Figure 4 shows the number of air pores in the concrete mixes with steel fibers (red line) and with polypropylene fibers (blue line) when compared to the reference concrete mix (dashed line).

Figure 4 shows the content of air-voids in the fresh concrete mixes with different types and contents of fibers. Generally, the mixes modified with the addition of fibers have more air-voids when compared to the reference concrete (even 40% more). Moreover, the content of air-voids in the mixes modified with fibers changes when the content of fibers increases (more air-voids). The concrete mixes with the polypropylene fibers had a smaller volume of air-voids when compared to the concrete mixes with steel fibers (an average difference of 0.75%). The addition of even 20 kg/m^3^ of steel fibers changes the content of air-voids in the concrete mixes by 2% when compared to the reference concrete. Similar results were observed in studies [37,38], in which the content of air-voids increased with an increasing amount of fiber in the mix. The authors noticed that the arrangement of fibers in the concrete is random, and therefore the formation of pores in the mix is also random. Small pores, such as capillary and gel pores, are formed in the matrix, and they have no relation to physical barriers (like fibers) [39]. In turn, steel fibers can generate larger bubbles in the concrete mix.

The differences in the content of air-voids in the concrete mixes with the addition of fibers are related to the fact that the addition of any material with a non-spherical shape causes an increase in the content of air-voids in the mixes. This is due to the fact that there is a reduction of the self-compacting properties of the mixes, which is caused by this additive (resulting from gravity forces). The distribution of air-voids and the orientation of the fibers inside the mix is affected by several parameters (geometry of the fibers, interaction effects: fibers-aggregates-formwork). Moreover, some researchers believe that the addition of steel fibers results in the formation of larger air-voids, which is due to the fact that these fibers are often contaminated. The highest content of air-voids in the mixes modified with steel fibers is related to the hooked-ends of these fibers. The polypropylene fibers have a different shape (continuous), and therefore do not have such a significant impact on the content of air-voids in the concrete mix when compared to the steel fibers.

#### 3.1.3. Bulk Density of the Concrete Mixes and Hardened Concrete

**Figure 5:**
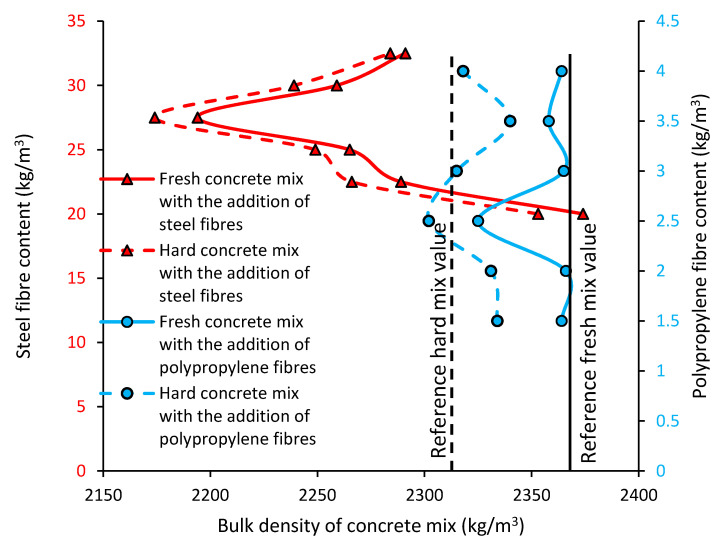
Bulk density of the concrete mixes modified with the addition of fibers.

Figure 5 presents the value of bulk density for the concrete mixes with the addition of steel (continuous red line) and polypropylene (continuous blue line) fibers when compared to the reference concrete (continuous black line). The value of the bulk density for the hardened concrete with the addition of steel fibers (dashed red line) and polypropylene fibers (dashed blue line), when compared to the reference hardened concrete (black dashed line), was also presented.

Figure 5 shows the bulk density of the fresh and hardened concrete with different types and contents of fibers. Generally, the fresh concrete modified with the addition of fibers has a similar value to the reference concrete. Moreover, the bulk density of the hardened concrete modified with fibers is lower than the bulk density of the fresh concrete. There is a nonsignificant relation between the addition of fibers and the bulk density of the concrete. All the obtained results are similar (with no more than 10% differences). Similar results were obtained in research [40,41]. The addition of steel or polypropylene fibers did not significantly change the bulk density of the concrete.

Slight changes in the bulk density of the fiber-modified concrete mixes are related to the low weight of the fibers when compared to the weight of the concrete (fiber weight is less than 1% of the weight of concrete). Evaporating water causes a greater change of density if fresh concrete and hardened concrete are compared. Such a change in the density is 3–4 times bigger than the change of density caused by fibers.

### 3.2. Determination of the Mechanical Properties of the Hardened Concrete

#### 3.2.1. Compressive Strength *f*_cm_ of the Destructive Tests of the Concrete

**Figure 6:**
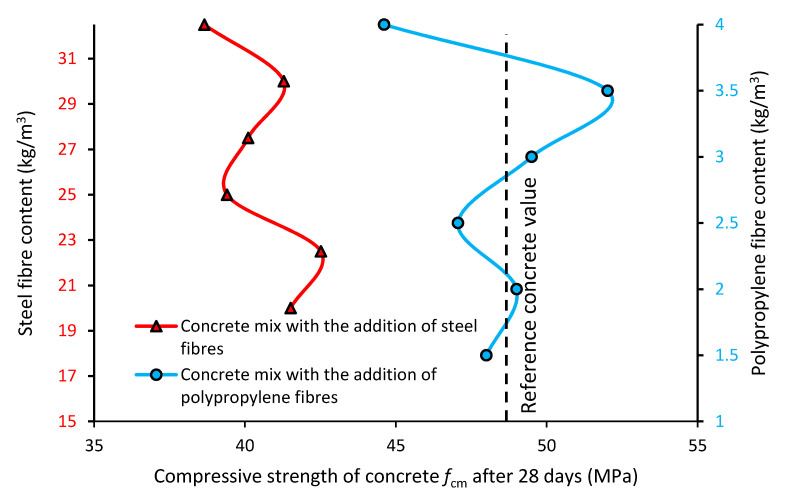
Compressive strength of the concrete modified with the addition of fibers.

Figure 6 presents the compressive strength *f*_cm_ of the concrete with the addition of steel (red line) and polypropylene (blue line) fibers when compared to the reference concrete (black dashed line).

Figure 6 shows the compressive strength *f*_cm_ of the concrete with different types and contents of fibers. The compressive strength of the concrete modified with the addition of fibers is lower than the strength of the reference concrete (even by 10%). Moreover, the compressive strength of the concrete modified with fibers does not significantly change when the content of the fibers increases. The compressive strength of the concrete with steel fibers is lower than the concrete without fibers (reference). The compressive strength of the concrete modified with polypropylene fibers is similar to the reference concrete. The hardened concrete modified with polypropylene fibers has a higher compressive strength than the concrete mixes with steel fibers (on average by 20%). The compressive strength of the concrete modified with polypropylene fibers is more or less stable (nonsignificant changes of ca. +/− 5%), but the compressive strength significantly decreases with the addition of these fibers in an amount higher than 3.5 kg/m^3^. The compressive strength of the concrete modified with steel fibers is also quite stable (nonsignificant changes of ca. +/− 5%), but the compressive strength significantly decreases with the addition of these fibers in an amount higher than 30 kg/m^3^. It can be seen that for both types of fibers, the addition of too many fibers causes a decrease in the compressive strength of the concrete. A significant increase in the compressive strength of the concrete (when compared to the reference concrete) was only observed in the case of the polypropylene fibers (with a content of 3–3.5 kg/m^3^). Steel fibers are most often added to concrete in an amount of 20–40 kg/m^3^. In these studies, the authors added the steel fibers in an amount of 20–32.5 kg/m^3^ in order to investigate the effect of an increased addition (a repeated increase by 2.5 kg/m^3^) of the fibers on the properties of concrete. The producers of polypropylene fibers indicate that in terms of the influence on the properties of concrete, the addition of 4 kg/m^3^ of these fibers corresponds to an addition of 35 kg/m^3^ of steel fibers. Based on this, the authors decided to investigate the addition of 1.5–4.0 kg/m^3^ of fibers (a repeated increase by 0.5 kg/m^3^) in order to compare the results obtained for both types of fibers.

In the research presented in paper [10], the results of 22 compressive strength tests were compared. It can be seen that the addition of steel fibers generally improves the compressive strength of concrete. In research [21], the addition of polypropylene fibers caused an increase in compressive strength (10%). Similar results were obtained in papers [22,41]. It should be noted that in the presented research, the authors could easily determine the apparent optimal amount of fiber added to the concrete. The authors of this article, unfortunately, did not achieve the apparent optimum dosing of fibers from a mechanical point of view. Unfortunately, there is no research on fiber-modified concrete, the composition of which is specially designed for the production of horizontally formed concrete.

Differences in the compressive strength observed in the case of adding both types of fibers may be related to the changes in consistency, which was induced by adding the fibers. Moreover, the random arrangement of fibers can cause the dissolution of research results. It may also be important that for the most optimal amount of fibers (30 kg/m^3^—steel fibers and 3.5 kg/m^3^—polypropylene fibers), there is a significant difference in the number of fibers of a particular type (for steel fibers with a content of 30 kg/m^3^—96 thousand pieces, for polypropylene fibers with a content of 3.5 kg/m^3^—462 thousand pieces) The compressive strength value is also dependent on the number of large air bubbles in the concrete. As shown in the literature [39], steel fibers can easily cause this type of bubble, which can reduce the compressive strength. A higher number of fibers can improve the compressive strength; however, this is the only hypothesis, and more research should be performed concerning this topic.

#### 3.2.2. Bending Tensile Strength *f*_ctm_ of the Concrete

**Figure 7:**
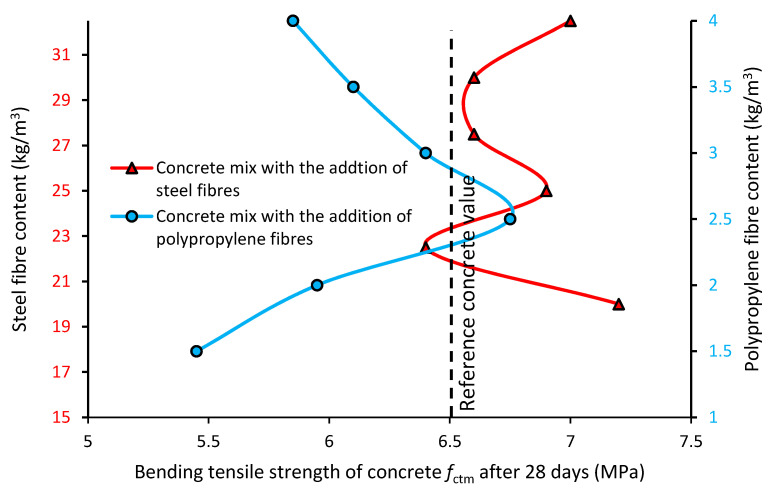
Bending tensile strength of the concrete modified with the addition of fibers.

Figure 7 presents the bending tensile strength *f*_ctm_ of the concrete with the addition of steel (red line) and polypropylene (blue line) fibers when compared to the reference concrete (black dashed line).

Figure 7 shows the bending tensile strength *f*_ctm_ of the concrete with different types and contents of fibers. Generally, the bending tensile strength of the concrete modified with the addition of fibers is comparable with the strength of the reference concrete. Moreover, the bending tensile strength of the concrete modified with fibers does not significantly change when the content of the fibers increases. The bending tensile strength of the concrete with steel fibers is higher (average 8%) than the concrete without fibers (reference). The bending tensile strength of the concrete modified with polypropylene fibers is lower (on average by 8%) than that of the reference concrete. The bending tensile strength of the concrete modified with steel fibers is higher than the bending tensile strength of the concrete mixes with polypropylene fibers (on average, by 16%). The bending tensile strength of the concrete modified with polypropylene fibers is variable (changes of ca. +/− 20%), but with the addition of fibers in an amount higher than 2.5 kg/m^3^, the bending tensile strength significantly decreases. The bending tensile strength of the concrete modified with steel fibers is quite stable (nonsignificant changes of ca. +/− 10%), but for testing the contents of the fibers (20–32.5 kg/m^3^), no optimal content was found. It can be seen that for the polypropylene fibers, the addition of too many fibers to the mix causes a decrease in the bending tensile strength of the concrete. A significant increase in the bending tensile strength of the concrete (compared to the reference concrete) was observed only for the 3.5 kg/m^3^ content of polypropylene fibers. Similar results were obtained in research [10,35]. As the addition of steel fibers increases, the bending tensile strength also increases. A significant effect of the hooked-ends of the steel fibers was also seen to improve the value of bending tensile strength. Different results were obtained in research [21,22], in which the bending tensile strength did not increase significantly with an increase in the addition of fibers.

The differences in the bending tensile strength observed for both types of fibers may be related to the diameter of the fibers. It is a fact that the pull-out strength of a single fiber is related to its diameter. The higher bending tensile strength value for the concrete modified with steel fibers can be related to the higher Young’s modulus value of these types of fibers. During the research, it was observed that the tested types of fibers have different destruction behavior. Bending tensile strength depends on the shape, length and type of fibers, but also on their interaction with the aggregate and matrix in the concrete. Concrete beams with the addition of steel fibers do not fall apart during destruction, and the fibers are not pulled out of the concrete. The opposite can be said for polypropylene fibers. During element destruction, polypropylene fibers are pulled out of the concrete, and the beam falls apart.

### 3.3. Brittleness B of the Concrete

**Figure 8:**
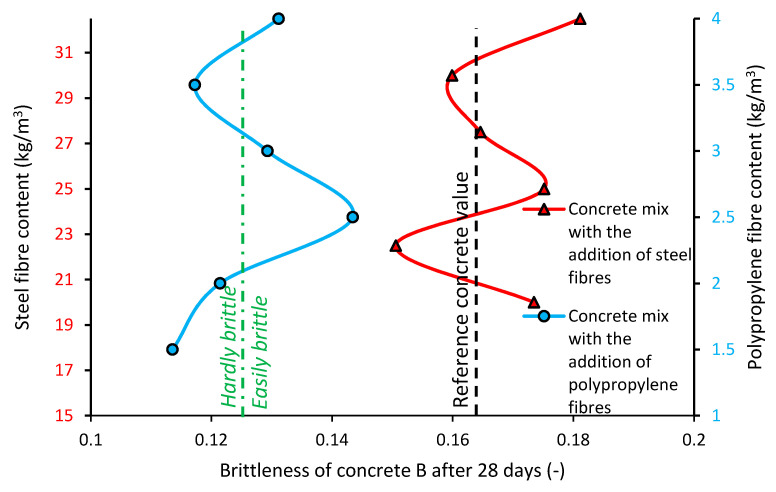
Brittleness of the concrete modified with the addition of fibers.

Figure 8 presents the brittleness B of the concrete with the addition of steel (red line) and polypropylene (blue line) fibers when compared to the reference concrete (black dashed line).

Figure 8 shows the brittleness B of the concrete with different types and contents of fibers. Generally, the brittleness value of the concrete modified with the addition of fibers is lower when compared to the brittleness of the reference concrete (even 20%). Moreover, the brittleness of the concrete modified with fibers does not depend on the content of the fibers. The brittleness value of the concrete with steel fibers is the same as the concrete without fibers (reference). The brittleness value of the concrete modified with polypropylene fibers is lower when compared to the reference concrete. The brittleness of the concrete modified with polypropylene fibers is lower when compared to the brittleness of the concrete modified with steel fibers (average 20%). It can be observed for the concrete modified with polypropylene that the average brittleness is low enough to be described as hardly brittle. On the other hand, the reference concrete and the concrete modified with steel fibers can be classified as easily brittle.

### 3.4. Correlations between the Properties of Concrete

Concrete properties are usually interrelated. This means that the mechanical properties of concrete (compressive strength, bending tensile strength) depend on the properties of the concrete mix (consistency, the content of air-voids). This influence must be especially considered in concrete with added fibers, as the fibers can influence these properties. It is well known that the shape, type and length of the fibers influence the properties of the concrete mix. The influence of the tested fibers on the properties of the concrete mix is presented below and compared with the properties of the hardened concrete.

**Figure 9:**
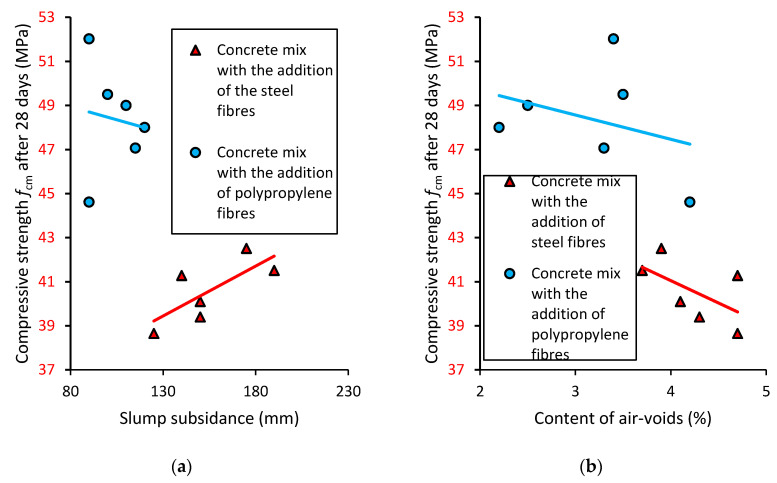
The relation between the compressive strength of the concrete modified with the addition of fibers, and (**a**) slump subsidence (**b**) content of air-voids in the concrete mixes.

Figure 9a shows the relationship between the slump subsidence value of the concrete mixes and the compressive strength of the concrete modified with different types and contents of fibers. Generally, it has been stated that the compressive strength of concrete depends on the consistency of the concrete mixes and the amount of water in the mixes. It can be seen that in the concrete modified with steel fibers, the increase in slump subsidence correlates to the increase in compressive strength. In the case of the concrete modified with polypropylene fibers, the increase in the value of slump subsidence correlates to the decrease in compressive strength. Figure 9b shows the relationship between the content of air-voids in the concrete mixes and the compressive strength of the concrete modified with different types and contents of fibers. Generally, it should be stated that the compressive strength of concrete is strongly related to the content of air-voids in the concrete mixes. It can be seen that in the concrete modified with steel and polypropylene fibers, the increase in the content of air-voids in the concrete mixes correlates to the decrease in the compressive strength of the concrete.

## 4. Mechanical and Economic Analysis of the Concrete Modified with Steel or Polypropylene Fibers

In order to analyze the properties of the concretes with the addition of steel and polypropylene fibers, their costs and mechanical properties were determined and then compared. In the mechanical analyses, the compressive strength and bending tensile strength were compared. In the economic analyses, the cost of preparing all the investigated series was calculated and compared.

### 4.1. Mechanical Performance Analysis

The mechanical performance of concrete is the most important criterion. To compare the mechanical performance of all the investigated concrete series, the mechanical performance ratio MPR was determined.

(2) $$\mathrm{MPR}=\frac{4\times \frac{f_{\mathrm{cm},\mathrm{mixes}\;}}{f_{cm,\mathrm{ref}}}+2\times \frac{f_{\mathrm{ctm},\;\mathrm{mixes}}}{f_{\mathrm{ctm},\mathrm{ref}}}}{6}\times 100\%\;\left(\%\right).\;$$

**Figure 10:**
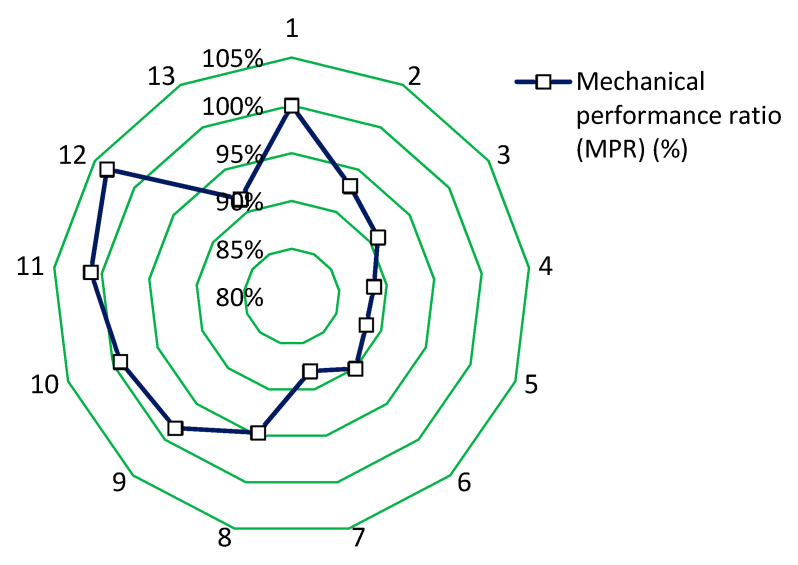
Mechanical performance ratio MPR of the concrete modified with the addition of fibers.

Based on the MPR determination, mechanical performance analysis was performed. The results of the analysis are shown in Table 2 and Figure 10.

Figure 10 shows the mechanical performance ratio MPR of the concrete with different types and contents of fibers. The mechanical performance ratio of the concrete modified with the addition of fibers is usually lower than the MPR value of the reference concrete (even by 13%). Moreover, the MPR value of the concrete modified with fibers does not significantly change when the content of fibers increases. The mechanical performance ratio value of the concrete with steel fibers is significantly lower than the concrete without fibers (reference). The mechanical performance ratio of the concrete modified with polypropylene fibers is similar to the value obtained for the reference concrete. The MPR value is higher for the concrete with polypropylene fibers, as is the case with the concrete with steel fibers (on average by 10%). The mechanical performance ratio values of the concrete modified with polypropylene fibers are stable (nonsignificant changes of ca. +/− 3%), which is similar to the concrete modified with steel fibers. It can be seen that the higher content of steel and polypropylene fibers in the concrete series causes the worsening of their mechanical properties (a clear optimal amount of both types of fibers).

### 4.2. Economic Performance Analysis

The economic performance of concrete is an important aspect that is often compared and analyzed. To compare the economic performance of concrete modified with steel or polypropylene fibers, economic performance analysis was performed. To analyze the economic properties, the effective cost ratio ECR was used. In the first step, the cost of all research series *C*_mixes_ was calculated.

(3) $$C_{\mathrm{mixes}}=c_{\mathrm{concrete}}+c_{\mathrm{fibres}}\;\left(€\right)$$

Based on the costs of concrete mixes, the effective cost ratio ECR was calculated.

(4) $$\mathrm{ECR}=\frac{C_{\mathrm{mixes}}}{C_{\mathrm{reference}}}\;\times 100\%\;\left(\%\right)$$

**Figure 11:**
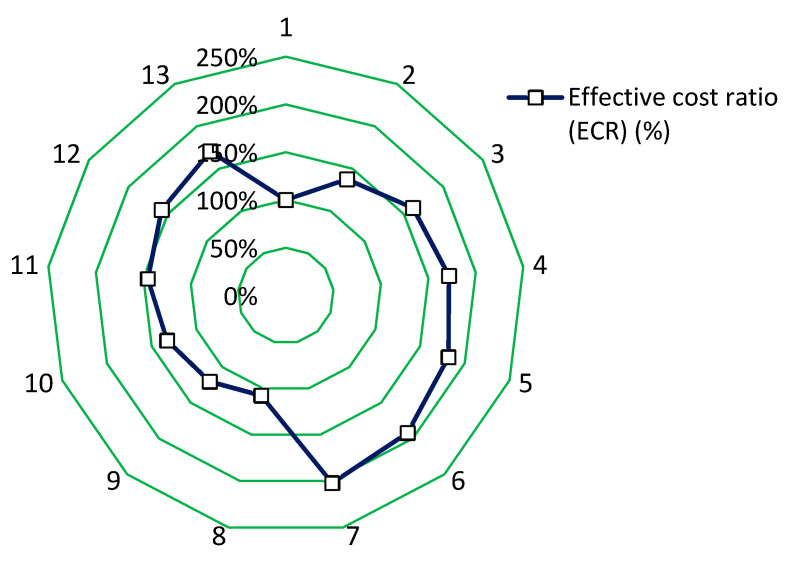
The effective cost ratio ECR of the concrete mixes modified with the addition of fibers.

The results of the economic performance analysis are shown in Table 3 and Figure 11.

Figure 11 shows the effective cost ratio ECR of the concrete with different types and contents of fibers. The effective cost ratio of the concrete modified with the addition of fibers is higher than the ECR value of the reference concrete (even by 200%). Moreover, the ECR value of the concrete modified with fibers does not significantly change when the content of fibers increases (for the used contents). The effective cost ratio value of the concrete with steel fibers is significantly higher than the concrete without fibers (reference). The effective cost ratio of the concrete modified with polypropylene fibers is also higher than the value of the reference concrete. The ECR value of the concrete with polypropylene fibers is lower than that of the concrete with steel fibers (on average by 50%). The effective cost ratio values of the concrete modified with polypropylene fibers increase when the content of fibers increases (ca. +50%), which is similar to the concretes modified with steel fibers. It can be seen that the higher content of steel and polypropylene fibers in the concrete series causes the worsening of their economic properties.

In research concerning steel fibers [42], it can be seen that the cost of the concrete mix is strongly related to the type and amount of added fibers. The cost of concrete series modified with fibers is significantly higher when compared to reference mixes (twice as much). The authors of the above-mentioned research also noticed that the cost of mixes modified with fibers depends on the type of used fibers (industrial or recycled steel fibers). Similar conclusions to those resulting from Table 3 were drawn in [20], in which the authors noticed that the use of polypropylene fibers, when compared to steel fibers, results in lower costs of concrete mixes. They also indicated that the addition of fibers to concrete mixes (added directly to the mixer) does not generate any extra manhours. Research [43] compared a few types of fibers. The authors noticed that the cost of concrete modified with fibers could be even 50% higher when compared to the reference concrete, but it can also be 20% lower.

Generally, it can be seen that the cost of steel fibers is related to their type (industrial or recycled), shape, dimensions and the season of the year. The cost of polypropylene fibers is not as variable as steel fibers. Using recycled fibers can reduce the cost of concrete. The cost of concrete mix ingredients (also fibers) depends on the part of the world and the access to raw materials.

### 4.3. Combined Economic and Mechanical Concrete Performance Analysis

The choice of concrete mix for construction elements should always be well thought out. The addition of fibers to the concrete mix could be an interesting idea for improving the properties of concrete; however, it should be stated that the addition of fibers to concrete mixes is quite expensive. In order to decide on the type and contents of added fibers, combined economic and mechanical concrete performance analysis was performed. The results of the analysis are shown in Table 4.

**Figure 12:**
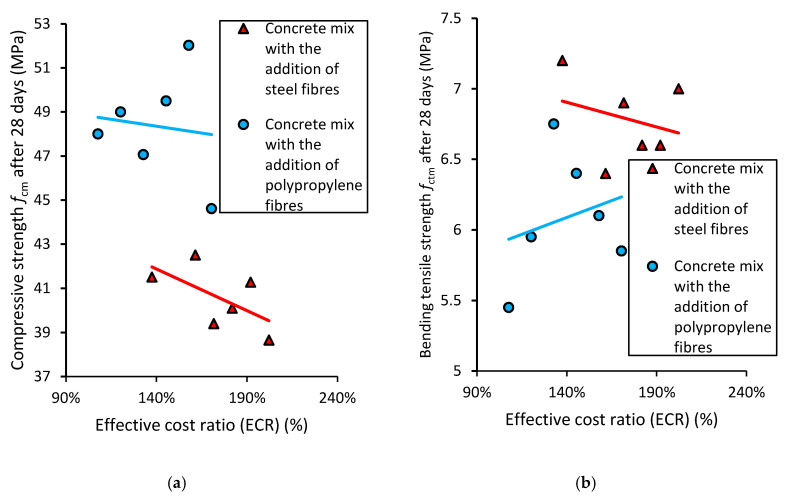
The relation between the effective cost ratio ECR and (**a**) compressive strength, (**b**) bending tensile strength of the concrete modified with the addition of fibers.

When summing up the results of the conducted analysis, two mechanical properties of concrete—compressive strength and bending tensile strength—were compared regarding the cost of the concrete—Figure 12.

Figure 12a presents the relation between the compressive strength of the concrete and the effective cost ratio ECR. The concrete modified with polypropylene fibers has better mechanical properties when compared to the concrete modified with steel fibers. Generally, it may be stated that a higher cost of concrete does not always correspond to higher mechanical performance. Figure 12b shows the relation between the bending tensile strength of concrete and the effective cost ratio ECR. The concrete modified with polypropylene fibers has worse mechanical properties when compared to the concrete modified with steel fibers. However, it can be seen that when the ECR increases (for mixes modified with polypropylene fibers), the mechanical performance of the concrete also increases. Overall, it can be stated that the higher costs of a concrete mix do not correspond with the higher mechanical properties of the concrete.

**Figure 13:**
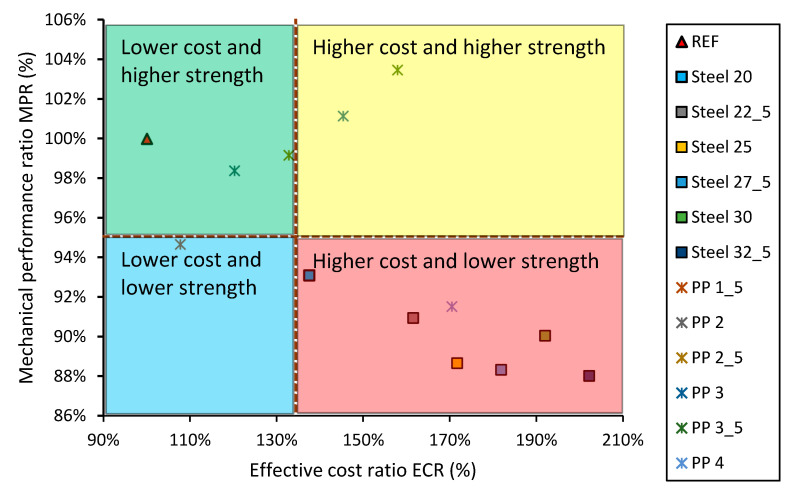
Comparing the types of concrete modified with the addition of fibers.

The performed analysis allows concrete mixes to be divided according to their mechanical and economic performance. Figure 13 shows the effect of this division.

Figure 13 shows the division of the concrete series modified with different types and contents of fibers into 4 different areas:

(1) Lower cost and higher strength, MPR ≥ 100% and ECR ≤ 130%;
(2) Higher cost and higher strength, MPR ≥ 100% and ECR ≥ 130%;
(3) Lower cost and lower strength, MPR ≤ 100% and ECR ≤ 130%;
(4) Higher cost and lower strength, MPR ≤ 100% and ECR ≥ 130%.

It can be seen that most of the series are in area 4 (the worst from the point of view of a designer), and none of the series show properties that would qualify it to be included in area 1 (the best from the point of view of a designer). The concrete modified with polypropylene fibers (5 series) has better properties when compared to the concrete modified with steel fibers.

## 5. Conclusions

The performed research and analyses allow the following conclusions to be drawn:

- The consistency of the fresh concrete mixes modified with the addition of polypropylene fibers is denser when compared to both the mixes modified with the addition of steel fibers and the reference mix,
- The addition of fibers to the concrete mix leads to an increase in the content of air-voids in the mix when compared to the reference mix. In the case of the analyzed changes in the dosage of fibers, the concrete mixes with the addition of steel fibers have a higher content of air-voids when compared to the concrete mixes with polypropylene fibers,
- The addition of fibers to the concrete mix does not cause significant changes in its bulk density,
- The compressive strength of the concrete modified with fibers is on a similar level or even lower when compared to the strength value of the reference mix. In the case of the analyzed changes in the dosage of fibers, the addition of the polypropylene fibers to the concrete mix leads to nonsignificant changes in the value of compressive strength, whereas the addition of steel fibers to the concrete mix leads to a decrease in compressive strength when compared to the reference concrete,
- The addition of steel fibers to concrete leads to an improvement in its bending tensile strength. For the amount of 2.5 kg/m^3^ of polypropylene fibers, an improvement of the concrete’s bending tensile strength when compared to the reference concrete was observed,
- The concrete modified with the addition of polypropylene fibers has better brittleness properties than the reference concrete and concrete modified with steel fibers,
- The addition of polypropylene fibers to concrete is economically much more sensible than the addition of steel fibers. However, it should be noted that both types of fibers have worse mechanical properties when compared to the reference concrete.

It should also be stated that the reason that fibers are added to concrete is the fact that they prevent the cracking of floors. For this dosage, civil engineers cannot expect any improvement in the compressive strength and bending tensile strength. To achieve this goal, higher contents should be implemented, which is not commonly used in concrete designed for floor purposes.

## Figures and Tables

**Table 1:** Series of concrete mixes.

Series	Fibers	Content
-	Type	kg/m^3^
REF	Reference	-
STEEL 20	Steel	20.0
STEEL 22_5	Steel	22.5
STEEL 25	Steel	25.0
STEEL 27_5	Steel	27.5
STEEL 30	Steel	30.0
STEEL 32_5	Steel	32.5
PP 1_5	Polypropylene	1.5
PP 2	Polypropylene	2.0
PP 2_5	Polypropylene	2.5
PP 3	Polypropylene	3.0
PP 3_5	Polypropylene	3.5
PP 4	Polypropylene	4.0

**Table 2:** Mechanical properties of the concrete modified with the addition of fibers.

Series	Fibers	Content	*f*_cm_	*f*_ctm_	MPR
-	Type	kg/m^3^	MPa	MPa	%
REF	Reference	-	45.68	7.38	100%
STEEL 20	Steel	20.0	41.51	7.2	93%
STEEL 22_5	Steel	22.5	42.51	6.4	91%
STEEL 25	Steel	25.0	39.4	6.9	89%
STEEL 27_5	Steel	27.5	40.1	6.6	88%
STEEL 30	Steel	30.0	41.28	6.6	90%
STEEL 32_5	Steel	32.5	38.65	7	88%
PP 1_5	Polypropylene	1.5	48	5.45	95%
PP 2	Polypropylene	2.0	49	5.95	98%
PP 2_5	Polypropylene	2.5	47.06	6.75	99%
PP 3	Polypropylene	3.0	49.5	6.4	101%
PP 3_5	Polypropylene	3.5	52.02	6.1	103%
PP 4	Polypropylene	4.0	44.61	5.85	92%

**Table 3:** Economic performance of the concrete mixes modified with the addition of fibers.

Series	Fibers	Content	*c*_concrete_	*c*_fibers_	*C*_mixes_	ECR
-	Type	kg/m^3^	€/m^3^	€/m^3^	€/m^3^	%
REF	Reference	-	48.00	0.00	48.00	100%
STEEL 20	Steel	20.0	48.00	18.00	66.00	138%
STEEL 22_5	Steel	22.5	48.00	20.25	68.25	162%
STEEL 25	Steel	25.0	48.00	22.50	70.50	172%
STEEL 27_5	Steel	27.5	48.00	24.75	72.75	182%
STEEL 30	Steel	30.0	48.00	27.00	75.00	192%
STEEL 32_5	Steel	32.5	48.00	29.25	77.25	202%
PP 1_5	Polypropylene	1.5	48.00	8.33	56.33	108%
PP 2	Polypropylene	2.0	48.00	11.11	59.11	120%
PP 2_5	Polypropylene	2.5	48.00	13.89	61.89	133%
PP 3	Polypropylene	3.0	48.00	16.67	64.67	145%
PP 3_5	Polypropylene	3.5	48.00	19.44	67.44	158%
PP 4	Polypropylene	4.0	48.00	22.22	70.22	170%

**Table 4:** Results of the combined economic and mechanical concrete performance analysis.

Series	Fibers	Content	MPR	ECR
-	Type	kg/m^3^	%	%
REF	Reference	-	100%	100%
STEEL 20	Steel	20.0	93%	138%
STEEL 22_5	Steel	22.5	91%	162%
STEEL 25	Steel	25.0	89%	172%
STEEL 27_5	Steel	27.5	88%	182%
STEEL 30	Steel	30.0	90%	192%
STEEL 32_5	Steel	32.5	88%	202%
PP 1_5	Polypropylene	1.5	95%	108%
PP 2	Polypropylene	2.0	98%	120%
PP 2_5	Polypropylene	2.5	99%	133%
PP 3	Polypropylene	3.0	101%	145%
PP 3_5	Polypropylene	3.5	103%	158%
PP 4	Polypropylene	4.0	92%	170%
